# Supplementary material for: Medical costs for patients with rheumatoid arthritis who have comorbid diabetes mellitus
Source: PLoS One. 2025 Aug 1;20(8):e0328094. doi: 10.1371/journal.pone.0328094 (PMC12316215; doi:10.1371/journal.pone.0328094)
Supplement: S1 Table — (PDF) [file pone.0328094.s001.pdf]

S1 Table. ICD-10 codes

Disease	ICD-10 codes
Included as RA	M050, M051, M052, M053, M058, M059, M060, M062, M063, M064, M068, M069
Included as DM	E10-, E100, E101, E102, E103, E104, E105, E106, E107, E109, E11-, E110, E111, E112, E113, E114, E115, E116, E117, E119, E12-, E13-, E130, E131, E132, E133, E134, E135, E136, E137, E139, E14-, E140, E141, E142, E143, E144, E145, E146, E149
Excluded as exclusion disease	D477, K500, K501, K508, K509, K510, K512, K513, K515, K518, K519, L400, L401, L402, L403, L404, L405, L408, L409, L732, M061, M080, M081, M082, M084, M088, M089, M303, M314, M316,

DM, diabets mellitus; ICD-10, International Classification of Diseases 10th Revision; RA, rheumatoid arthritis
