# Supplementary material for: Medical costs for patients with rheumatoid arthritis who have comorbid diabetes mellitus
Source: PLoS One. 2025 Aug 1;20(8):e0328094. doi: 10.1371/journal.pone.0328094 (PMC12316215; doi:10.1371/journal.pone.0328094)
Supplement: S3 Table — (PDF) [file pone.0328094.s003.pdf]

S3 Table. Items included in drug costs for treatment of comorbidities

Drug type	Ingredient name
Antidiabetic drugs	Acarbose, Acetohexamide, Alogliptin benzoate, Alogliptin Benzoate/Metformin Hydrochloride, Alogliptin Benzoate/Pioglitazone Hydrochloride, Anagliptin, Anagliptin/Metformin Hydrochloride, Buformin hydrochloride, Canagliflozin hydrate, Chlorpropamide, Dapagliflozin Propylene glycol hydrate, Dulaglutide, Empagliflozin, Empagliflozin/linagliptin, Exenatide, Glibenclamide, Gliclazide, Gliclopyramide, Glimepiride, Insulin aspart, Insulin degludec, Insulin Degludec/Insulin Aspart, Insulin degludec/liraglutide, Insulin detemir, Insulin glargine, Insulin glulisine, Insulin human, Insulin lispro, Ipragliflozin L-proline, Linagliptin, Liraglutide, Lixisenatide, Luceogliflozin hydrate, Metformin hydrochloride, Miglitol, Mitiglinide calcium hydrate, Mitiglinide Calcium Hydrate/Voglibose, Nateglinide, Omarigliptin, Pioglitazone hydrochloride, Pioglitazone hydrochloride/glimepiride, Pioglitazone hydrochloride/metformin hydrochloride, Repaglinide, Saxagliptin hydrate, Sitagliptin phosphate hydrate, Sitagliptin phosphate hydrate/ipragliflozin L-proline, Teneligliptin hydrobromide hydrate, Teneligliptin hydrobromide hydrate/canagliflozin hydrate, Tofogliflozin hydrate, Trelagliptin
Antihypertensive drugs	Acebutolol hydrochloride, Alacepril, Alanidipine, Aliskiren fumarate, Amlodipine besilate/atorvastatin calcium hydrate, Amlodipine besylate, Amosulalol hydrochloride, Arotinolol hydrochloride, Atenolol, Azelnidipine, Azil sultan, Azilsartan/amlodipine besylate, Benazepril hydrochloride, Benidipine hydrochloride, Bentyl hydrochlorothiazide, Bentyl hydrochlorothiazide/reserpine combination, Betaxolol hydrochloride, Bevantolol hydrochloride, Bisoprolol, Bisoprolol fumarate, Bunazosin hydrochloride, Candesartan cilexetil, Candesartan cilexetil/amlodipine besylate, Candesartan cilexetil/hydrochlorothiazide, Captopril, Carteolol hydrochloride, Carvedilol, Ceriprolol hydrochloride, Cilazapril hydrate, Cilnidipine, Clonidine hydrochloride, Delapril hydrochloride, Diltiazem hydrochloride, Doxazosin mesylate, Efonidipine hydrochloride ethanol adduct, Enalapril maleate, Eplerenone, Esaki selenone, Felodipine, Guanabenz acetate, Hydralazine hydrochloride, Hydrochlorothiazide, Imidapril hydrochloride, Indapamide, Irbesartan, Irbesartan/amlodipine besylate, Irbesartan/Trichlormethiazide, Labetalol hydrochloride, Lisinopril hydrate, Losartan potassium, Losartan potassium/hydrochlorothiazide, Manidipine hydrochloride, Mefluside, Methicrane, Methyldopa hydrate, Metoprolol tartrate, Nadolol, Nicardipine hydrochloride, Nifedipine, Nilvadipine, Nipradilol, Nisoldipine, Nitrange pin, Olmesartan medoxomil, Olmesartan medoxomil/azernidipine, Perindopril Erbumin, Pinroll, Prazosin hydrochloride, Propranolol hydrochloride, Quinapril hydrochloride, Reserpine, Spironolactone, Telmisartan, Telmisartan/amlodipine besylate, Telmisartan/amlodipine besylate/hydrochlorothiazide, Telmisartan/hydrochlorothiazide, Temocapril hydrochloride, Terazosin hydrochloride hydrate
Antidyslipidemic drugs	Amlodipine besilate/atorvastatin calcium hydrate, Arilocumab, Atorvastatin calcium hydrate, Bezafibrate, Cholestyramine, Clinofibrate, Clofibrate, Colestimide, Dextran sulfate sodium, Elastase, Ethyl icosapentate, Evolocumab, Ezetimibe, Ezetimibe/atorvastatin calcium hydrate, Ezetimibe/rosuvastatin calcium, Fake troll, Fenofibrate, Fluvastatin sodium, Gamma oryzanol, Lomitapidomesylate, Nico Mall, Omega-3 fatty acid ethyl, Pantetin, Pema fibrate, Pitavastatin calcium, Polyenephosphatidylcholine, Pravastatin sodium, Probucol, Rosuvastatin calcium,
