# Supplementary material for: Medical costs for patients with rheumatoid arthritis who have comorbid diabetes mellitus
Source: PLoS One. 2025 Aug 1;20(8):e0328094. doi: 10.1371/journal.pone.0328094 (PMC12316215; doi:10.1371/journal.pone.0328094)
Supplement: S6 Table — (PDF) [file pone.0328094.s006.pdf]

S6 Table. List of excluded medical cares.

Main Category	Subcategory	Sub-subcategory	Medical List Group Code	Medical ID	Medical Name
Initial and Follow-up Fees	Initial and Follow-up Fees	Initial Consultation Fee	A000	111013370	Pregnancy Addition (Initial)
		Follow-up Consultation Fee	A001	112021370	Pregnancy Off-hour Addition (Follow-up, Outpatient)
				112022070	Pregnancy Addition (Follow-up)
Hospitalization Fees	Basic Hospitalization Fee Additions	Emergency Hospitalization for Pregnant Women Add-on	A205-3	190126910	Emergency Hospitalization for Pregnant Women Add-on
	Short-stay Surgery Basic Fees	Short-stay Surgery Basic Fee	A400	190179210	Short Surgery Type 3 (Lens Reconstruction and Insertion)
Tests	Laboratory Test Fees	Urine/Feces Testing	D004	160060910	General Semen Test
	Physiological Test Fees	Examinations Using Monitoring Devices	D218	160073410	Labor Monitoring over 1h30m
Procedures	Procedure Fees	Obstetric and Gynecologic Procedures	J072	140015210	Vaginal Irrigation
			J073	140015410	Uterine Cavity Irrigation
			J077	140015810	Uterine Hemostasis during Delivery
			J079	140016210	Cauterization of Cervical Portion
			J080	140016310	Laminaria Insertion
				140016610	Metreurynter Insertion
			J082	140017110	Non-invasive Correction of Uterine Prolapse (Pessary)
Surgeries	Surgery Fees	Eye	K282	150253010	Lens Reconstruction with Intraocular Lens (Other)
				150356210	Lens Reconstruction with Suture-fixed Lens
			K282-2	150280650	Secondary Cataract Surgery
Surgeries	Surgery Fees	Genital	K893	150221210	Vacuum Extraction Delivery
			K898	150222210	Cesarean Section (Elective)
			K909	150224110	Abortion Surgery (up to 11 Weeks)
			K911	150224410	Hydatidiform Mole Removal
Anesthesia	Anesthesia Fees	Anesthesia Fees	L009	150333370	Cesarean Section Anesthesia Add-on
