# Supplementary material for: Medical costs for patients with rheumatoid arthritis who have comorbid diabetes mellitus
Source: PLoS One. 2025 Aug 1;20(8):e0328094. doi: 10.1371/journal.pone.0328094 (PMC12316215; doi:10.1371/journal.pone.0328094)
Supplement: S7 Table — (PDF) [file pone.0328094.s007.pdf]

S7 Table. Sensitivity analysis of total medical cost by matching ratio of non-DM group against DM g

Type of medical cost	Matching ratio	Medical costs per patient, n, mean (SD), USD/year				
		DM		Non-DM		*P*-value
Including DM costs	1:1	109	6,347 (5,234)	109	3,810 (4,050)	< 0.001
	1:2	80	6,153 (5,363)	160	3,797 (4,271)	< 0.001
	1:3	62	6,105 (5,393)	186	3,688 (3,766)	< 0.001
Excluding DM costs	1:1	109	5,163 (5,017)	109	3,782 (4,018)	< 0.001
	1:2	80	4,893 (5,069)	160	3,765 (4,235)	0.005
	1:3	62	4,942 (5,061)	186	3,653 (3,720)	0.010
