# Supplementary material for: Medical costs for patients with rheumatoid arthritis who have comorbid diabetes mellitus
Source: PLoS One. 2025 Aug 1;20(8):e0328094. doi: 10.1371/journal.pone.0328094 (PMC12316215; doi:10.1371/journal.pone.0328094)
Supplement: Attachment — Submitted filename: response_to_reviewers_CQ1_27May2025_resptorev.docx [file pone.0328094.s009.docx]

Dear Reviewers,

Thank you for your careful review of our manuscript and for your constructive comments. We have revised the manuscript according to your suggestions and would be grateful if you could review the updated version.

Sincerely,

Eiichi Tanaka

**Reviewer #1**

***Comments to the Authors***

*The study is very interesting and the results obtained have been satisfactorily discussed. Further studies would be needed that take into account the limitations of the current study, as clarified by the Authors themselves.*

**Author Response:** Thank you for the comment. We appreciate you taking the time to review our manuscript.

**Reviewer #2**

***Comments to the Authors***

*The study is well written and benefits from stringent inclusion criteria regarding ICD10 codes. Also, the limitations of the study have been thoroughly addressed in the last sections. The limitation to data availability has also been adequately justified. I would like to point out a few points that might need some additional explanation or revisiting:*

**Author Response:** Thank you for the comment. We have updated our manuscript according to your suggestions as follows.

***Comment 1***. *Lines 214-216: when assessing treatment costs you specified some exclusions, such as cataract operations. Would it be possible, if achievable, to provide a list of the excluded costs (maybe in a supplementary table) or, alternatively, declare the general criterion behind the choice of excluding some costs?*

**Author Response:** Thank you for the comment. We have added the exclusion criteria and a list of excluded medical cares.

Methods section (Page 13, lines 214-217)

To assess treatment and material costs, we considered all costs for medical care during the follow-up period (shown in S4 and S5 Tables S4 and S5), with some exceptions, such as cataract operations and childbirth-related medical care (shown in S4 and S6 Tables).

***Comment 2***. *Lines 365-366: "NSAIDs are commonly used in patients with RA, but patients often require higher doses to reduce inflammation". This affirmation, while being plausible according to clinical experience, seems to be lacking solid evidence in literature from comparative studies. In the absence of a citation that may back this statement, I would suggest to rephrase or delete the sentence, if not necessary for the narrative of your discussion.*

**Author Response:** Thank you for your comment. We have deleted the texts as follows.

Discussion section (Page 29, lines 366-367)

NSAIDs are commonly used in patients with RA, but patients often require higher doses to reduce inflammation.

***Comment 3***. *Finally, the list of supplementary tables at the end of the document mentions a table S6: Sensitivity analysis of total medical cost by matching ratio of non-DM group against DM group, which apparently was not included in the downloadable xlsx file.*

*Overall, the article is well-structured and it will certainly provide useful insights of the topic after incorporating the suggested revisions.*

**Author Response:** Thank you for your comment. We gratefully apologize for the lack of the table. We have added as S6 Table.
